# Development of the Movement Pattern Observation Tool (MPOT)—An Observational Tool to Measure Limb Movements during Elementary School Recess

**DOI:** 10.3390/ijerph20085589

**Published:** 2023-04-20

**Authors:** Gemma Kate Webb, Deborah J. Rhea

**Affiliations:** Kinesiology Department, Texas Christian University, Fort Worth, TX 76129, USA

**Keywords:** unilateral, bilateral, contralateral, limb movements, unstructured play, recess

## Abstract

Background: The US Center for Disease Control estimates that only 24% of American elementary-aged children participate in the recommended 60 min of daily physical activity. As activity levels decline, elementary schools should consider increasing movement opportunities. Activity-driven school days, where children can move their limbs freely, may increase memory retention performance, behavioral impulse control, as well as bone density, and muscle strength. Unstructured, outdoor play (recess) may provide an opportunity for the brain, bone, and muscle-stimulating limb movements to be utilized. To date, no research has focused on whether the modern child actively uses limb movements during recess, nor to what degree. The purpose of this study was to develop a reliable assessment tool (Movement Pattern Observation Tool, MPOT) to observe and record limb movements (unilateral, bilateral, and contralateral movements) of elementary children during recess, defined in this study as unstructured, outdoor play. Methods: Three observers used the MPOT to complete thirty-five observations at one elementary school during kindergarten through fifth-grade recess breaks. Results: Interrater reliability approached excellent, being that excellent is above 0.90. The ICC of the master observer and observer 3 value was 0.898 (95% CI 0.757–0.957), and the ICC of the master observer and observer 2 was 0.885 (95% CI 0.599–0.967), *p* < 0.03. Conclusion: Inter-rater reliability was achieved through a three-phase process. This reliable recess observation tool will contribute to the body of research linking recess to physical and cognitive health.

## 1. Introduction

The US Center for Disease Control estimates that only 24% of American elementary-aged children participate in the recommended 60 min of daily physical activity [1]. Childhood inactivity is detrimental to brain, muscle, and bone development [2]. Elementary-age children are in a crucial developmental stage where physical activity habits can help or hinder proper bone density and muscle strength for years to come [3]. Limb movements, produced by physical activity (PA), create opportunity for the skeletal and muscular systems to overload, aiding proper development, increasing motor skill acquisition, and decreasing chances of injury [4,5]. Regardless of their aerobic or anaerobic metabolic demands, limb movements during PA create stimulation and maturation in two brain regions: (1) the hippocampus for memory retention; and (2) the prefrontal cortex for attention and impulse control [6]. Active children, therefore, have advantages over inactive children in physical and cognitive domains through limb movements. As activity levels continue to decline for elementary-aged children, elementary schools should consider increasing movement opportunities within the school day. Activity-driven school days, where children can move their limbs freely, may increase performance in memory retention, and behavioral impulse control, as well as increase bone density and muscle strength. Increasing recess in schools, defined in this study as unstructured, outdoor play, may provide an opportunity for this brain, bone, and muscle-stimulating limb movements to be utilized.

### 1.1. Theoretical Framework: Bartenieff Fundamentals and Fundamental Movement Skills

An eight-week-old fetus moves in distinct motor patterns that are easily recognizable and continue after birth [7]. It is through these patterned limb movements that the body and brain can correctly develop [8]. These patterns deserve special attention as advancement or alterations in limb movement patterns are a reliable indicator of brain (dys)function in a developing fetus and baby [4]. Mastery of these movement patterns continues to develop through adulthood and is necessary for the correct development of brain networks to emerge [9].

Bartenieff Fundamentals (BF) [10] is a theoretical framework adopted by disciplines such as dance to describe fundamental body movements. Bartenieff Fundamentals consists of six movement patterns, including three limb movements (unilateral, bilateral, and contralateral) and three other involuntary movements (breath, naval radiation (core-distal), and spinal movement (head-tail). This framework specifies that these movements begin in utero and continue as varying forms throughout the lifecycle, emphasizing the importance of making connections within our bodies through these movements [10]. By utilizing these patterns throughout life, individuals can achieve more comfortable, efficient, pain-free, and enjoyable movements [8] They have also been shown to help improve executive function [11].

Studies with this framework confirm unilateral, bilateral, and contralateral movements as fundamental limb movements of the human body throughout the lifecycle and necessary for the proper development of the body and the brain as humans develop from birth throughout adulthood [7,8,9]. Utilizing these basic limb movements prepares the body, brain, and nervous system to complete more complex tasks as the body matures. In connection with BF, using limb movements during unstructured childhood play creates movement efficiency, allowing for mastery of more advanced movements known as Fundamental Movement Skills (FMS). FMS encompasses traditional functional movement skills (kick, run, jump, throw, leap, dodge, and catch) as well as combined skills (resistance training movements, riding a bicycle, or swimming strokes) [5]. Successful participation in FMS during childhood is associated with higher physical activity levels throughout the lifecycle [12]. Conversely, if a certain level of FMS competence is not achieved in early childhood, a child’s development of future motor skills will be hampered, leading to less lifelong activity pursuits and increasing risk of all-cause mortality [5,13]. Limb use during physical activity and free play may directly influence lean muscle mass development and movement experience, which are essential for the successful completion of any movement task [14]. The benefits of unilateral, contralateral, and bilateral limb movements can produce a positive compounding effect as children utilize one side of the body, or alternating use of both sides of the body, to create new neuropathways [15], making light work of both simple and complex movements. As nerve connections are built, reaction times improve, and the body can quickly and more efficiently recruit muscles to perform tasks. As the American culture is becoming increasingly obesogenic, children are at risk for heightened sedentary lifestyles [1]. One assertive approach to combating sedentary behaviors is recess, defined in this study as an unstructured outdoor play within the school setting. Recess may be an essential gateway for limb movement utilization in children. A great deal of research has investigated the benefits of utilizing and mastering FMS. However, to date, no research has observed and quantified the rudimentary limb movements (unilateral, bilateral, and contralateral) needed for success in FMS among elementary-aged children, nor to what extent limb movements are found during recess.

### 1.2. Gap in Current Assessment Tools

Current assessments exist to test strength in unilateral or bilateral movements, but no known assessment exists to examine the quantity of unilateral, bilateral, and contralateral movements during recess. Assessments such as The Functional Movement Screen [16] and the Foundational Movement Skills Tests [17] have been used to measure movement competencies required for proficient participation in physical activity. No current assessment exists to determine how often limb movements are utilized during childhood activities such as recess. This information is necessary as limb movements create the muscle and brain development needed to accomplish the foundational and functional movements required for physical activity participation and advanced motor skill proficiency.

Accelerometers are another assessment used to measure moderate and vigorous physical activity (MVPA) and steps during play. They consist of a wristband device that children/students wear for a designated time, and then removed for analysis of MVPA and steps through appropriate software. Although accelerometers successfully determine how active a child is during play, they are limited by recording only the speed of movements and the number of steps. When a child is hanging by two arms or legs from a bar, significant muscle, bone, and brain-to-muscle neuro-developments occur within the body, but the accelerometers will read that as no motion or steps. Accelerometers give a picture of how frequently limb movements occur, aiding in assessing whether a child is increasing cardiovascular fitness during the monitored activity or not. Accelerometers do not account for the muscle-brain strengthening movements that require slower limb activation, and therefore, fall short of determining whether the child is increasing bone, muscle, and neurological efficiency through the monitored activity. Though much accelerometer research focuses on child activity levels during recess, no tool exists to show the specific limb movements utilized during recess, nor to what degree. Therefore, the purpose of this study was to develop a reliable assessment tool, the Movement Pattern Observation Tool or MPOT, to quantify unilateral, bilateral, and contralateral movements of elementary children during school recess and determine the Interrater reliability of this assessment tool.

## 2. Materials and Methods

An average of 495 participants spanning all phases of the assessment tool development consisted of kindergarten, first-grade, and second-grade students among two different school districts in North Central Texas. Five elementary schools were used to collect a variety of observations during recess. The schools and districts utilized were chosen due to the accessibility of locations to the research team and the approval of administration and principals to observe recess in the schools. This study began in September 2021 with research on baseline limb movements utilized during physical activity. Preliminary recess observations began in October 2021 to determine if these movements can be recorded during recess. The creation and modification of the MPOT were from October 2021 through May 2022. The overall study from start to finish was eight months. Three MPOT development phases were completed using the five schools randomly to confirm that this assessment tool was viable to use on playgrounds during recess with elementary school children. For the reliability of the MPOT, interrater reliability was calculated for the MPOT with Interclass Correlation Coefficients (ICC) and their 95% confidence intervals using SPSS statistical package version 28. The results were based on a mean rating (k = 2), absolute-agreement, and a two-way mixed-effects model. Thirty-five observations from the three observers were used to determine this result.

Interrater reliability was calculated using Interclass Correlation Coefficients (ICC) and their 95% confidence intervals for each observation comparison of the MPOT using SPSS (IBM statistical package for Macintosh, version 28.0). The results were based on a mean rating (k = 2), absolute-agreement, and a two-way mixed-effects model. ICC values less than 0.5 are indicative of poor Reliability, values between 0.5 and 0.75 indicate moderate reliability, values between 0.75 and 0.9 indicate good reliability, and values greater than 0.90 indicate excellent Reliability [18,19,20]. Thirty-five total observations were conducted between observer 1 (expert rater) and two other observers to test the Interrater Reliability.

### 2.1. Phase 1: Identifying the Movements Observed in Recess

The purpose of Phase 1 was to study the types of activities children participated in at recess and categorize them into unilateral, bilateral, contralateral, and “no movement” categories. To accomplish this task, two researchers observed four elementary school recesses consisting of first- and second-graders to document any physical movements observed. These grades were chosen because the students fit the elementary student criteria and based on the recess times available for the researchers to observe. A scan from left to right was conducted of the entire play area as all activities observed were recorded by hand. Hash marks were used to note repeated movement activities. Examples of activities recorded included skipping, sliding, hanging on bars, moving across the monkey bars, kicking or dribbling a soccer ball, using a jump rope to jump, climbing up a climbing wall, and running. The left-to-right scan was continued for the entirety of the scheduled 20 min recess. Once two researchers observed four recesses each, the listed activities were accumulated and analyzed. Each activity was grouped into unilateral, bilateral, contralateral, or no-movement categories. All recorded activities fell into one of the four categories; therefore, the three limb movements were confirmed as the complete list of limb movements to assess during recess. With this established, an observation tool and protocol were created next to efficiently capture and record the limb movements of children during recess.

### 2.2. Phase 2: Development of a Limb Movement Assessment Tool

**Figure 1:**
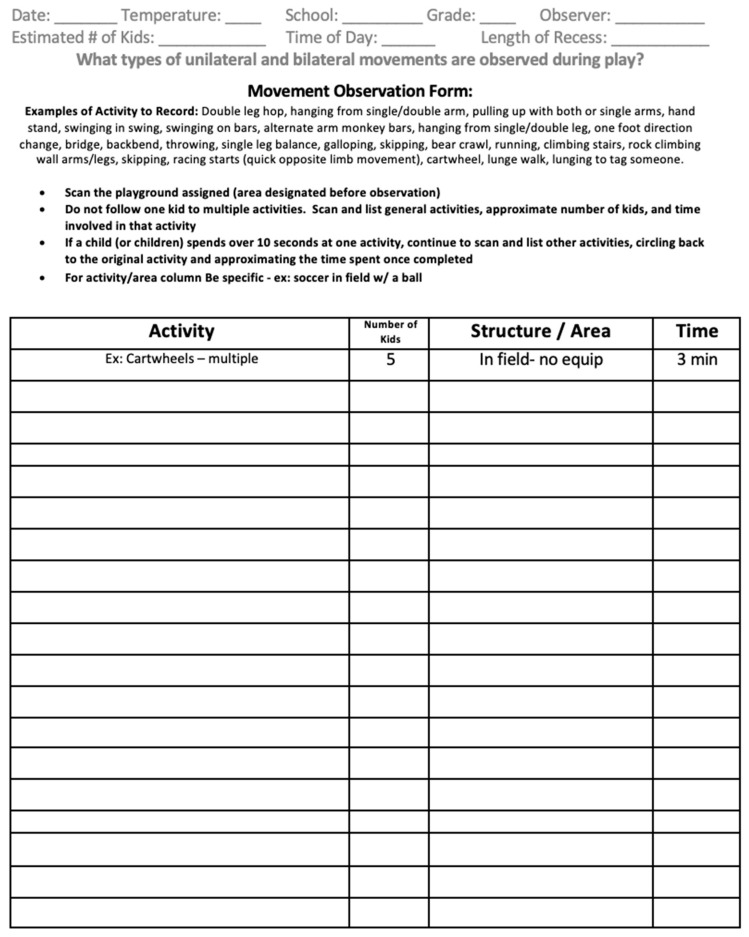
First Movement Pattern Observation Tool.

Phase 1 led to the validation of the different activities and limb movements observed on the playground above and the creation of the first iteration of this new tool named the Movement Pattern Observation Tool (MPOT). The first MPOT (Figure 1) was designed for two observers to scan one-half of the playground each. Each observer would write the movements they observed in the chart. This tool was used to observe one elementary school recess and the recordings were analyzed. The analysis showed that the form did not capture what the individual child was participating in but rather what equipment or section of the playground was getting the most use. For example, the monkey bars may have been traversed thirty times within 10 min, with thirty hash marks, but the same five children may have completed the thirty traverses.

**Figure 2:**
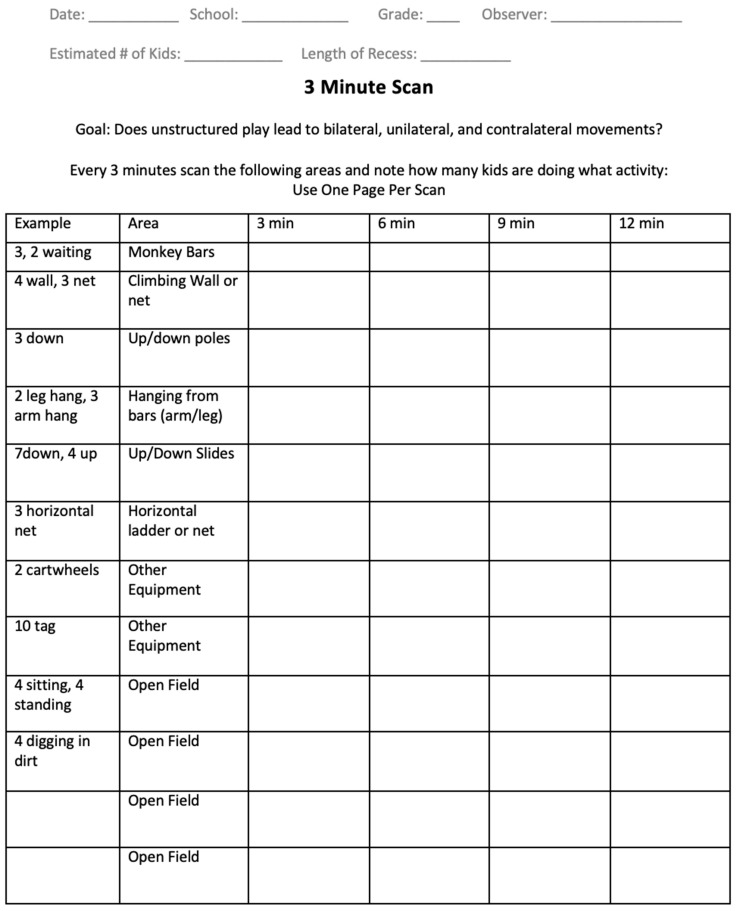
Second Movement Pattern Observation Tool.

Therefore, revisions were made to the first iteration, and a second version of the MPOT (Figure 2) was created to capture the individual activity per child with a three-minute “snapshot” scan. This “snapshot” format was included to observe the entire play area within three minutes. Three minutes were chosen through trial and error in the field. Two minutes did not allow enough time to complete observations, while five minutes seemed too long. A snapshot scan was accomplished by observing a designated section of the play area and scanning that section from left to right, spending no more than 20 s per section. A specific recording pattern was established among structures, swing sets, and open field areas for time efficiency. The observer recorded all activities seen as if a photo was taken of the scanned section. If the child moved from one activity to another within the designated section, the observer recorded the greater of the two movements.

The head researcher used this second MPOT version for two elementary school recess periods on two separate days for a total of four observations. The use and ease of the form were analyzed. The primary benefit of the snapshot scan style of recording was that it allowed the observer to traverse the entire play area efficiently, allowing more individual students to be observed. Although some students moved from one play area to another during observations, less repetition of the same children was observed with this format, and a greater percentage of the children were recorded with the different types of activities than with the original form. Although the snapshot scan produced a more accurate method of recording individual movements, another modification of the form design was necessary to create efficiency of commonly recorded activities.

Modifications for the final observation tool stemmed from the feedback of a new group of researchers/observers (focus group) to create an observation tool that would be usable and efficient across various researchers for future study purposes. The first focus group consisted of two new observers whom the master observer trained in the use of the MPOT, and then they used the MPOT at one elementary school for three different grade-level recesses in one day. The observers gave feedback after the observations were completed which helped to determine efficient recording mechanisms and column construction. The most common activities recorded in all previous tools were noted and grouped into categories to be noted with a hash mark instead of writing.

Once the MPOT modifications were made, the second focus group, comprising the same two observers and the lead researcher, observed three recesses (kindergarten, first-grade, and second-grade) from one school in one day. After each recess, the group discussed any questions about usability and suggestions for the tool’s efficiency or the observation instructions. Modifications from their feedback included grouping the activity categories by the limb movements required, moving “run” to the top of the form since it was the most commonly used activity, and differentiating between gender within each category. The three, three-minute scans were modified to two, four-minute scans. Although the lead researcher could complete the observations within three minutes due to many familiarities, the focus team needed closer to four minutes due to inexperience in labeling the movements. As one uses the tool more often, efficiency can increase, but for training and usability purposes, four minutes were more appropriate. Finally, standing had been recorded in earlier MPOT versions as bilateral activity, but through further discussion, standing was shifted to the non-movement category. Though standing creates a bilateral load on the musculoskeletal system, this study is focused on limb movements and the neurological development that occurs when utilizing them, hence the label change.

**Figure 3:**
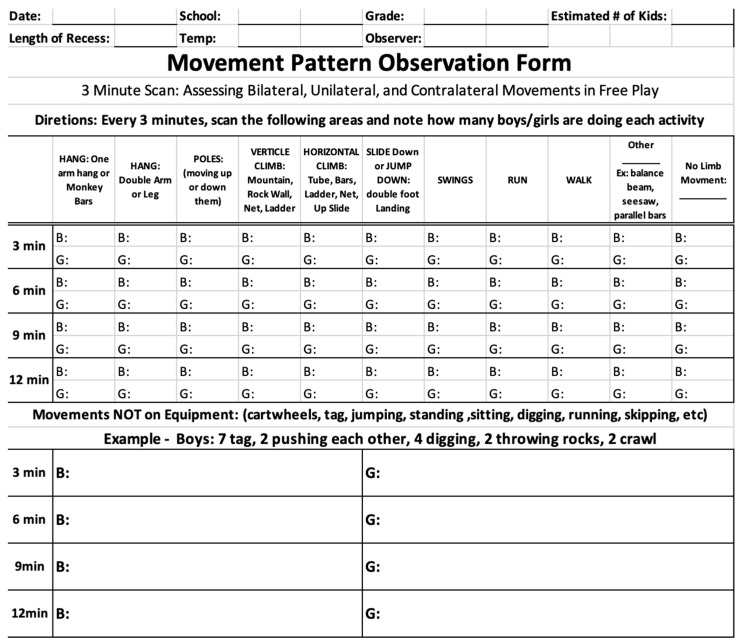
Final Movement Pattern Observation Tool (MPOT).

The MPOT final version (Figure 3) was constructed from the observer focus group feedback given above. The master observer practiced observations with the final MPOT version in two or more kindergarten, first-grade, and second-grade 15 min recesses. Within each school district, two MPOT observations were completed per kindergarten, first- and second-grade, for a total of 6 observations per district and 12 observations overall. The number of children observed ranged from 90–130 per recess, depending on the grade level of the participating school. One school averaged 90–100 children per grade level, whereas the other averaged 110–130 children per grade level. Eighty-three percent of the total children attending recess were observed with the MPOT. The other 17% were not captured as they may have been transitioning from one playground section to another. After each observation, the master observer analyzed the data by categorizing the activities into unilateral, bilateral, contralateral, and no-movement categories. The total number for each category was recorded in Excel for each grade and recess individually to gather raw data totals. From the master observer’s perspective, the final observation tool was easy to use, captured the children in different areas of the play spaces, and quickly identified unilateral, bilateral, and contralateral movements throughout each recess period.

### 2.3. Phase 3: Interrater Reliability of the MPOT

The tool was now ready for interrater reliability analysis through the completion of multiple observations, focus group feedback, and the development of the final MPOT version in phase two. Thirty-five observations of kindergarten through fifth-graders were completed to test the interrater reliability in May 2022. This sample population was chosen due to the school principal’s approval to allow recess observations The master observer and two other trained observers (observer 2 and observer 3) participated in the interrater reliability assessment at an elementary school where none of the three observers had previously assessed. All three observers are part of a research project called LiiNK (Let’s inspire innovation ‘N Kids). LiiNK researchers work with school districts to increase the amount of time allotted for daily recess, defined as unstructured, outdoor play (60 min) and daily 15 min character lessons (Positive Action). LiiNK also provides three full-day training to prepare teachers and administrators for the intervention [21]. The three observers collected both observational data, such as classroom and recess observations, as well as body fat percentages, hair cortisol samples, and attentional focus testing. The two observers completed a 30 min training with the master observer during one school recess before the observations began. During that training, the master trainer explained how to use the MPOT, followed by several rounds of 3 min scans where the master trainer orally described how they were using the form. After the explanation and trial runs, the two observers orally described what they were observing so the master trainer could hear. They discussed any discrepancies and the master trainer answered questions. Finally, all three observers silently completed one three-minute round of observations and then compared notes and answered any further questions. The master trainer emphasized the importance of noting the particular limb movements, not the successful completion of a skill. For example, the MPOT is designed to note whether the student is using only one of the four limbs, two upper limbs, two lower limbs, or opposite and opposing limbs during the snap-shot scan. Whether or not the student successfully completes a pull-up on a bar, lands a jump, or skips is irrelevant. The observer is just to notate if limbs are utilized, and if so, which category of the three it fell into. Both observers showed through discussion and oral practice attempts and then through silent observation that they were ready to use the MPOT for recess observations. During observations, each of the observers was blinded to the other observers by creating a broad proximity between observers. Clipboards were also used by observers and held in a position where others could only see the back of the clipboard, not the recorded data. The master observer and observer 2 used the MPOT for 23 four-minute comparison observations, and the master observer and observer 3 used the MPOT for 12, four-minute comparison observations. Statistical analyses were run to determine internal consistency between observers. As evidenced below, the difference in the number of observations between observers did not appear to impact the interrater reliability, possibly alluding to reliable use without extensive practice.

## 3. Results

Table 1 shows the ICC of the master observer and observer 3 value was 0.898, and the ICC of the master observer and observer 2 was 0.885 (*p* < 0.03). Therefore, based on statistical inference, it was concluded that the reliability is “good” approaching “excellent.”

Table 2 shows the actual numeric comparisons of each limb movement, total limb movements, and those not participating in limb movements between each set of observers. In twelve observations, observer 1 captured 163 children in total, and observer 2 captured 176 children in total. In 23 observations, observer 1 captured 325 children at play, and observer 3 captured 326 children at play. It is evident that the observers showed the same trends in the amount of activity observed. Observers 1 and 2 comparisons showed contralateral to be by far the most commonly used limb movement, followed by bilateral movements, no movement, and then unilateral movements, respectively. Observers 1 and 3 comparisons showed contralateral to be the most dominantly utilized movement, followed by not moving, bilateral movements, and unilateral movements, respectively. Although the number of children moving vs. non-moving ranked a close second or third overall among the observers between the four movement categories, the combined total of children moving limbs compared to those not moving limbs when observed was exponentially higher. Observers 1 and 2 comparisons showed 80–83% of children participated in one of the three types of limb movements when observed, while observers 1 and 3 comparisons showed 70–78% of the children participated in one of the three limb movements.

## 4. Discussion

Activities such as school recess have been studied in the realm of moderate to vigorous activity, heart rate, and cardiovascular conditioning. However, until the creation of the MPOT tool, no research existed to record limb movements of the modern child during recess, nor to what degree. With the development and reliability of the final MPOT, it is now possible to assess elementary children’s unilateral, bilateral, and contralateral and “no” movements during school recess. This tool is an important assessment, as limb movements aid in developing proper skeletal and muscular strength and neurological pathways for efficient movement and brain development. The benefits of unilateral, contralateral, and bilateral limb movements can produce a positive compounding effect as children utilize one side of the body, or alternating use of both sides of the body, to create new neuro-pathways [15] making light work of both simple and complex movements. This not only increases the health and wellness of the child but stimulates the brain through nerve stimulation and oxygen consumption, prepping the students for academic focus upon returning to the classroom.

The data in Table 2 points to the MPOT accuracy, noting trends in movement patterns as well as showing what percentage of children are actively recruiting limbs during recess compared to those not utilizing their limbs. All thirty-five observations show the children participating in recess were utilizing contralateral movements more than the other two movements combined. Although this needs to be explored further, this trend has major implications for recess advocacy during school days. Contralateral movements (such as running, crawling, climbing) recruit the entire body, opposite and opposing limbs, and the limbs often cross the anatomical midline. This mass movement and midline crossing has been shown in research to aid in neuromuscular and cognitive development [7], and correlates with improved attention and concentration within the classroom [22].

All thirty-five observations had uniform category groupings among the two observers. For instance, observers 1 and 2 had bilateral movements as the second most utilized movement and no limb movement as the third, whereas observers 1 and 3 had no limb movements ranking second and bilateral movements ranking third. This adds to the MPOT reliability, and although the specific numbers vary, the trends are still very similar and evident among the observers.

Another noteworthy finding in Table 2 is that out of over three-hundred students, observers 1 and 3 were only off by one student in total students observed. Observers 1 and 2 were only off by thirteen students in the 173 students observed. Children quickly shifted from one activity to the next within the given scanned area, leading to a slight variation in observations, but the total number of children observed was very close in each category and overall, considering the massive number of children observed.

Children who were standing with no other limb activity were placed into the nonmovement category. This is due to the limbs not actually moving, although it could be debated that a load is being placed upon the musculoskeletal system while standing. Children who were standing during these observations were often in groups or circles of kids and seemed to be planning their next move. Very often a circle of standing children would all turn and run right after observations. Other standing circles seemed to be female students visiting. Since socialization is very important to children, and especially females as they age, many were taking the opportunity to socialize while standing or walking.

### Limitations

Observers that aided the master observer had previous experience with classroom and recess observations. This may have aided in the ease of training, and therefore, the thirty-minute training may not be adequate for observers using the MPOT with no previous observation experiences. Another limitation is that all observations were performed at one school. Future studies with more variety in schools and play areas will be needed. Another limitation is the validity of MPOT was not assessed and the ICC interrater reliability was assessed after only thirty-five observations. Although this is a limitation, Table 2 shows that a large number of students were observed. The descriptive data show similar notations of limb movements, as well as similar trends in most commonly used to least commonly used movements. These data increase the confidence in using the MPOT despite these limitations.

A threat to internal validity was possible since children could be observed more than once during the snap-shot scan due to children constantly traveling across the playground. To combat this internal threat, the four-minute observation window allowed the observer to quickly move from section to section to limit overlapping children observed. The set data collection method of the equipment first, and then the area around the equipment, the open field, and finally the swings, was also to quicken the observation and keep a consistent flow in each observation scan.

## 5. Conclusions

The MPOT is found to be a reliable tool to observe which limb movements are being utilized by children during recess and to what degree. In addition to the ICC reliability of the MPOT being “good, approaching excellent,” the information in Table 2 shows the consistency between observers across the limb movement categories. Although the number of students participating in each activity is not exact across observers, the general number of kids participating in each limb movement or no limb movement was similar across all categories for each group of observers. The baseline trends showing contralateral as the most commonly used limb movement, as well as 70% or more of all children observed participating in limb movements, are very impactful for the health and wellness of American children. This is at a time in our history when most children are categorized as having inactive daily lifestyles. Continued studies of the MPOT will enhance the existing body of research that shows recess as a viable means for increasing activity levels in children and offsetting the detriments of sedentary lifestyles.

The goal of this study was to capture limb movement patterns during recess. Since the MPOT was found to be capable of capturing this, larger and more varying populations should be studied to generalize findings in future studies. Future studies with the MPOT may include determining the most commonly used limb movement(s) during recess, how many children utilize limb movements during recess depending on grade level or gender, and when children begin to decrease their limb activities based on grade level and gender.

## Figures and Tables

**Table 1:** ICC of MPOT.

Interclass Correlation Coefficient					
			95% Confidence Interval		
Average Measures	N	Intraclass Correlation	Upper Bound	Lower Bound	Sig
Master Observer and Observer 2	23	0.898	0.757	0.957	<0.001
Master Observer and Observer 3	12	0.885	0.599	0.967	<0.001

**Table 2:** Summary of Movement Patterns Observed.

Total Movements between Observers							
Number of Observations	Observers	Unilateral	Bilateral	Contralateral	Total Kids Moving Limbs	Total Kids Not Moving Limbs (# Standing)	Total Kids Observed
12	Observer 1	19	30	86	135 (83%)	28 (19)	163
	Observer 2	8	35	98	141 (80%)	35 (22)	176
23	Observer 1	36	66	150	252 (78%)	73 (61)	325
	Observer 3	37	72	119	228 (70%)	98 (87)	326

#: Number.

## Data Availability

Availability of data and materials: All data and materials are available upon request from G. Kate Webb at g.kate.webb@tcu.edu.
